# The value of folate receptor‐positive circulating tumor cells in the diagnosis of lung cancer and its correlation with clinical characteristics

**DOI:** 10.1111/crj.13601

**Published:** 2023-03-28

**Authors:** Yunjian Xu, Qianjun Li, Zhijian Lin, Yongping Lin

**Affiliations:** ^1^ Department of Clinical Lab The First Affiliated Hospital of Guangzhou Medical University Guangzhou China

**Keywords:** biomarker, clinical characteristics, diagnosis, folate receptor‐positive circulating tumor cells, lung cancer

## Abstract

**Objective:**

The aim of this research is to investigate the feasibility of folate receptor‐positive circulating tumor cells (FR+CTCs) as a biomarker for the diagnosis of malignant pulmonary nodules and the correlation between clinicopathological factors and FR+CTC levels.

**Methods:**

Patients initially diagnosed with one or more pulmonary nodules from a computed tomography scan were prospectively included. Three milliliters of peripheral blood was collected from each participant for FR+CTC analysis prior to surgery. Clinical and pathological parameters and FR+CTC levels were compared between patients with lung cancer and benign diseases.

**Results:**

Six hundred fifty‐three patients had lung cancer and the other 124 had benign lung diseases based on pathological examinations of the resected specimens. The median FR+CTC value of the lung cancer group was 12.0 (95% CI 9.6–16.2) FU/3 mL and that of the benign group was 7.2 (95% CI 5.78–11.2) FU/3 mL. The difference was statistically significant (*P* < 0.0001). In a receiver operating characteristic analysis to distinguish the two groups, the area under curve of FR+CTC was 0.7457 (95% CI 0.6893–0.8021; *P* < 0.0001) using a cutoff of 8.65 FU/3 mL. The sensitivity was 86.37%, and the specificity was 74.19%. Combined with conventional serum tumor biomarkers, the area under curve was 0.922 (0.499–0.963). The sensitivity was 92.20%, and the specificity was 83.05%. FR+CTC levels were related to tumor staging (*P4* < 0.001), the degree of tumor invasion both in single (*P* = 0.011) and multiple lesions (*P* = 0.022), pathological subtypes (*P* = 0.013), and maximum tumor diameter (*P* = 0.014).

**Conclusions:**

FR+CTC is an effective and reliable biomarker for the diagnosis of lung cancer. Further, FR+CTC level is correlated with tumor staging, degree of invasion, pathological subtypes, and tumor size.

## Graphical abstract

FR+CTC is an effective and reliable biomarker for the diagnosis of lung cancer. Combining conventional serum tumor biomarkers, including CA125, CEA, CYFRA21‐1, CA153, and NSE, could improve diagnostic efficiency. Furthermore, FR+CTC level is correlated with tumor staging, degree of invasion, pathological subtypes, and tumor size.

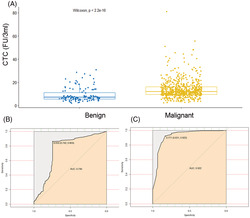

## 1 INTRODUCTION

Lung cancer is the second most common malignancy worldwide according to the latest survey; in China, lung cancer is the most and the second most prevalent cancer type in males and females, respectively, and it also has the highest mortality rate among all cancer types in both genders.^1^, ^2^, ^3^ Early diagnosis is key in lung cancer management.

Currently, lung cancer is primarily diagnosed using imaging techniques. Computed tomography (CT) examination can increase the rate of auxiliary diagnosis of lung nodules, but sometimes CT‐assisted diagnosis of focal ground‐glass opacity is misdiagnosed because of low visibility. Certain serological tumor markers are also used for lung cancer screening and diagnosis. The most commonly used ones include squamous cell carcinoma (SCC) antigen, carcinoma embryonic antigen (CEA), cytokeratin 19 fragment antigen 21‐1 (CYFRA21‐1), and neuron‐specific enolase (NSE). Studies have shown that these tumor markers can be used for in diagnosis, prognosis, and follow‐up surveillance of lung cancer.^4^, ^5^, ^6^ However, these tumor markers suffer from low sensitivity especially in early‐stage lung cancers, thereby limiting their use in early diagnosis.^7^ In practice, identifying new biomarkers for early diagnosis of lung cancer is highly desired.

Recent studies have suggested that the vascular invasion of tumor cells may happen as early as at the precancerous stage, leading to the release of circulating tumor cells (CTCs) into the circulatory system.^8^, ^9^ Thus, detection of such CTCs may help early diagnosis and management of lung cancer patients.^10^ In the last decade, technology advancements have enabled reliable detection of CTCs in clinical practice.^11^ Because peripheral blood specimens are easy to obtain, less invasive, and can be repeatedly sampled, CTCs may be a promising new biomarker for routine clinical assessment.^12^ Folate receptor‐α has been extensively studied as a potential target for cancer diagnosis and therapy. In the peripheral blood, only a few FR‐expressing cells are present, including CTCs and a rare subtype of activated monocytes which are seldom detected in nonmalignant individuals.^13^, ^14^ Further, FR is generally not expressed in normal blood cells except a rare type of activated macrophages.^15^

Several studies have demonstrated the utilities of folate receptor‐positive CTCs (FR+CTC) in diagnosis and prognosis of lung cancer and a few other cancer types that are known to have high FR expressions.^16^, ^17^, ^18^ For lung cancer diagnosis, the sensitivity and specificity of FR+CTC were reported to be 72.5–81.8% and 82.4–93.2%, respectively.^16^, ^17^ However, the diagnostic value of FR+CTC combined with other tumor biomarkers has not been well explored, and the correlation between FR+CTC and other conventional clinical and pathological factors remain to be explored.

The primary goal of this study was to investigate the value of FR+CTCs combined with traditional tumor biomarkers for the diagnosis of benign and malignant pulmonary nodules. In addition, the correlation between FR+CTC and other conventional clinical and pathological factors was explored.

## 2 MATERIAL AND METHODS

### 2.1 Study design

This is a prospective, observational study to investigate FR+CTC in early diagnosis of lung cancer and the association between FR+CTC and the clinicopathological characteristics of lung cancer patients. From March 2019 to May 2020, 777 patients who were diagnosed with one or more pulmonary nodules based on CT scan were enrolled at the First Affiliated Hospital of Guangzhou Medical University. The study was approved by the Research Ethics Committee of the First Affiliated Hospital of Guangzhou Medical University. All patients provided written informed consent before inclusion into this study.

### 2.2 Inclusion and exclusion criteria

Inclusion criteria are the following: (1) ages between 18 and 80 years and Eastern Cooperative Oncology Group performance status of 0–1; (2) scheduled to receive surgical resection upon physician's assessment; (3) no previous antitumor therapy (including preoperative neoadjuvant therapy); and (4) informed consent to collect peripheral blood for FR+CTC detection before surgery. All participants were required to fast overnight before blood collection. The exclusion criteria were the following: (1) patients with other severe diseases and not suitable for tissue biopsy or surgical treatment; (2) a history of other malignancies within the past 5 years, except for cancer that had been cured and had no risk of recurrence or metastasis; (3) incomplete patient information; (4) patients with leukocyte counting >1.2 × 10^7^/mL or <2 × 10^6^/mL (as extreme white blood cell count could affect the enumeration of CTCs); (5) patients who took folic acid tablets long term and stopped taking <3 days prior to obtaining the blood sample (as high folic acid concentration has reported to lower the expression of FR); and (6) abnormalities during sample collection or processing such as inadequate amount of blood, blood clots in the sample, hemolysis, incomplete lysis of red blood cells, adhesion of the sample cells after lysis, or insufficient amount of cells after lysis.

### 2.3 Definition

Lung cancer staging was based on the 7th IASLC TNM Staging System. Benign lung diseases included hamartoma, pneumonia, tuberculosis, intrapulmonary lymph node, bronchiectasis, and granuloma. According to IASLC/ATS/ERS classification of lung adenocarcinoma (ADC), ADC in situ (AIS) is defined as non‐invasive ADC showing no stromal, vascular, or pleural invasion, whereas minimally invasive ADC (MIA) and invasive ADC (IAC) are defined as AIC containing an invasive component.^19^

### 2.4 CTC enrichment, labeling, and quantification

FR+CTC analysis was performed using CytoploRare® kit provided by GenoSaber Biotech Co. Ltd. (Nantong, China). According to the manufacturer's instruction, CTCs were negatively enriched where erythrocytes were lyzed by lysis buffer and leukocytes were depleted using anti‐CD45 immunomagnetic beads. Next, CTCs were incubated with a proprietary probe that consists of conjugates of a folic acid analog and a synthesized oligonucleotide. Then, the unbound probes were washed off. The bound probes were removed with stripping buffer and collected by centrifugation and neutralized.

FR+CTC was quantified by quantitative polymerase chain reaction analysis. First, the probe was annealed and extended before amplification. Then, the extended probe was analyzed using a Taqman probe on ABI StepOne™ system (Thermo Fisher, Waltham, MA, USA). The primer sequences and reaction conditions were as described by Lou et al.^20^ “CTC unit” in this study represents the number of CTCs detected in 3 mL of blood, for example, 10 CTC units stands for 10 CTCs in 3 mL of blood. It should be noted that as a PCR‐based CTC detection method, background noise could lead to the virtual positive value which did not exactly represent the presence of CTCs. Only FR+CTC levels higher than the determined cutoff threshold are considered to be positive for FR+CTC. A serial of standards containing oligonucleotides ranging from 10^−14^ to 10^−9^ M is used for CTC quantification, which corresponds to 2–2 × 10^5^ CTC U/3 mL blood. Samples from each patient were tested in duplicates with six standards and three quality controls.

### 2.5 Tumor biomarker tests

Serum CTCs were collected prospectively, and cancer antigen 125 (CA125), CEA, cytokeratin 19 fragment (CYFRA21‐1), cancer antigen 153 (CA153), NSE, and SCC antigen were collected retrospectively. Tumor biomarkers including CA125, CEA, CYFRA21‐1, CA153, and NSE were tested by electrochemical luminescence (ECLINA) (Roche, Rotkreuz, Switzerland). SCC antigen was tested by a chemiluminescent microparticle immunoassay using the ARCHITECT I2000 SR system (Abbott, Axsym, Ireland).

### 2.6 Statistical analysis

Statistical analysis was performed using the R software and the Prism 5.0 system (GraphPad Software Inc., San Diego, CA, USA). Wilcoxon test, Mann–Whitney test, and Kruskal–Wallis test were used for comparison between groups as appropriate. *P* < 0.05 was considered to indicate a statistically significant difference. Receiver operating characteristic (ROC) curve was used to examine the diagnostic efficiency as indicated by the area under the ROC curve. Youden index was used to determine the optimal cutoff threshold. Logistic regression was used to establish a joint diagnostic model of different biomarkers for distinguishing lung cancer from benign lung diseases. Pearson's correlation was used to analyze the association between FR+CTC levels and clinical factors.

## 3 RESULTS

### 3.1 Patient characteristics

Seven hundred seventy‐seven patients were included in this study, of which 653 had lung cancer and 124 had benign lung diseases. The median age was 58 and 55 years old, respectively. There were 321 males and 332 females in malignant cohort and 77 males and 47 females in benign cohort. Of the 653 lung cancer patients, 77.79% of those had ADC, 58 (8.89%) had SCC, and the remaining 87 had other lung cancer types. The 57.1% of the lung cancer was Stage I. The detailed characteristics are shown in Table 1.

**TABLE 1:** Characteristics of lung cancer and benign diseases.

Characteristics	Number	Ratio (%)
Lung cancer (*n* = 653)		
Age, years (median, range)	58 (49–65)	
Gender (male)	321	49.16
Female	332	50.84
Tumor stage		
AIS	24	3.7
I	373	57.1
II	38	5.8
III	30	4.6
IV	24	3.7
Unsureness	33	5.1
Histopathologic subtype		
ADC	508	77.79
SCC	58	8.89
Others	87	13.32
Benign diseases (*n* = 124)		
Age, years (median, range)	55 (44.2–64)	
Gender (male)	77	62.10
Female	47	37.9

Abbreviations: ADC, adenocarcinoma; AIS, adenocarcinoma in situ; SCC, squamous cell carcinoma.

### 3.2 Diagnostic value of FR+CTCs

**FIGURE 1:**
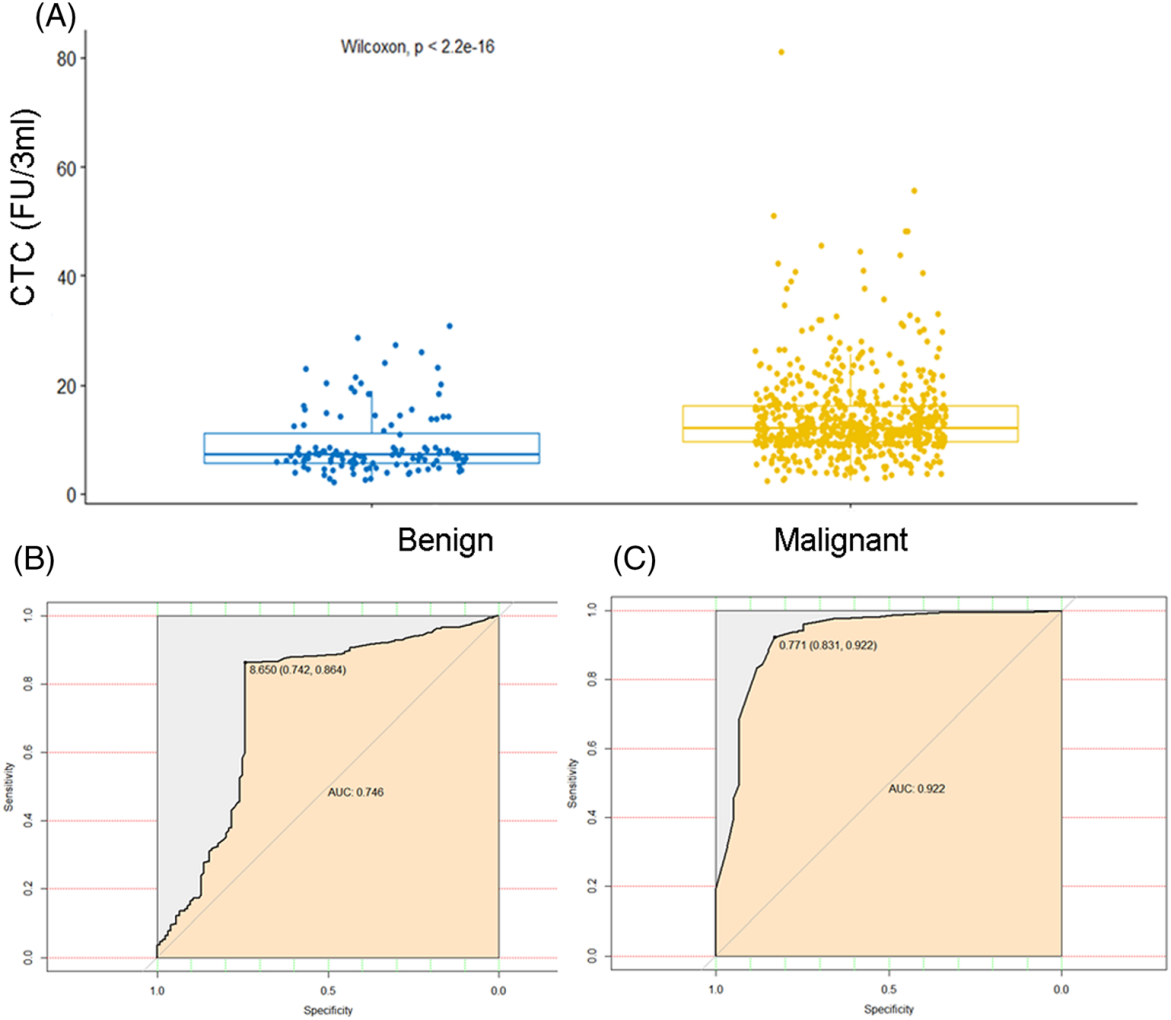
Diagnostic value of folate receptor‐positive circulating tumor cells (FR+CTCs). (A) FR+CTC levels in lung cancer and noncancer participants; (B) receiver operating characteristic curve for FR+CTC; (C) receiver operating characteristic curve for combined FR+CTC with neuron‐specific enolase, carcinoma embryonic antigen, cancer antigen 125, cancer antigen 153, and cytokeratin 19 fragment antigen 21‐1.

We first compared the FR+CTC levels between patients with lung cancers and benign participants. As shown in Figure 1A, the median FR+CTC level of the lung cancer group was 12.0 FU/3 mL (interquartile range 9.6 to 16.2) and that of the benign group was 7.2 FU/3 mL (interquartile range 5.78 to 11.2). The difference in the FR+CTC level between the two groups was statistically significant (*P* < 0.0001). The 8.65 FU/3 mL was selected as the optimal cutoff threshold for differentiating lung cancer from noncancer patients, and the sensitivity and the specificity of FR+CTC was 86.37% and 74.19%, respectively. The area under curve of FR+CTC in differentiating lung cancer from benign diseases was 0.746 (95% CI 0.689–0.802; *P* < 0.0001) (Figure 1B). Meanwhile, the diagnostic performance of NSE, CEA, CA125, CA153, CYFRA21‐1, and SCC was evaluated; the sensitivities of these serum biomarkers ranged from 26.19% to 86.0%. FR+CTC exhibited the best performance (*P* = 0.0001) in terms of sensitivity in differentiating patients with lung cancer from benign participants. It was similar to that of CA153 and outperformed traditional serum tumor biomarkers (NSE and CA125) twofold to threefold (Table 2). The performance of FR+CTC combined traditional serum tumor biomarkers (except of SCC) was 0.922 (0.499–0.963) (Figure 1C).

**TABLE 2:** Comparison of the diagnostic value between FR+CTC and tumor biomarkers.

	Controls	Patient	AUROC (95% CI)	*P*	Cutoff	Sensitivity	Specificity
FR+CTC	124	653	0.746 (0.689–0.802)	0.0001	8.650 FU/3 mL	86.37% (564/653)	74.19% (92/124)
NSE	59	205	0.537 (0.451–0.622)	0.4004	18.880 ng/mL	40.98% (84/205)	47.56% (28/59)
CEA	100	503	0.627 (0.572–0.682)	0.0001	2.405 ng/mL	47.91% (241/503)	76.00% (76/100)
CA125	99	501	0.530 (0.468–0.592)	0.3487	15.625 U/mL	34.13% (171/501)	55.56% (55/99)
CA153	99	500	0.499 (0.440–0.559)	0.9957	6.430 U/mL	86.0% (430/500)	7.07% (7/99)
CYFRA21‐1^a^	59	205	0.640 (0.565–0.715)	0.0002	3.365 ng/mL	45.37% (93/205)	79.66% (47/59)
SCC	5	13	0.731 (0.500–0.963)	0.0515	0.850 ng/mL	53.85% (7/13)	100% (5/5)
FR^+^CTC + NSE + CEA + CA125 + CA153 + CYFRA21‐1	59	205	0.922 (0.499–0.963)	<0.0001	0.7714	92.20% (189/205)	83.05% (49/59)

Abbreviations: AUROC, area under the ROC; CA125, sugar chain antigen CA125, CA125; CA153, sugar chain antigen CA153, CA153; CEA, carcinoma embryonic antigen; CYFRA21‐1, cytokeratin 19 fragment antigen; FR+CTC, folate receptor‐positive circulating tumor cell; NSE, neuron‐specific enolase; ROC, receiver operating characteristic; SCC, squamous cell carcinoma antigen.

^a^ The data of CYFRA21‐1 are for squamous cell carcinoma.

### 3.3 Clinical factors associated with FR+CTC levels

The correlations between FR+CTCs and clinical pathological characteristics were analyzed to explore the application of FR+CTCs for lung cancer. All the information of lung cancer groups is summarized in Table 3. Five hundred eight out of the 653 lung cancer patients with ADC had data, 58 cases were SCC, and the median FR+CTC level in SCC group is 13.80 FU/3 mL, which was higher than the group of ADC (11.60 FU/3 mL); there was a statistical difference between the two groups (*P* = 0.013). Four hundred eighty‐nine out of the 653 lung cancer patients had tumor staging data after surgical resection. Of these patients, 24, 373, 38, 30, and 24 had AIS, Stage I, Stage II, Stage III, and Stage IV tumors, respectively. As shown in Table 3, the level of FR+CTC increased with the progression of tumor stage. The median level was 10.45 FU/3 mL for AIS, 11.40 FU/3 mL for Stage I, 11.95 FU/3 mL for Stage II, 12.75 FU/3 mL for Stage III, and 18.10 FU/3 mL for Stage IV (*P* = 0.00037).

**TABLE 3:** Correlation of FR+CTC levels and clinical characteristics in lung cancer.

Characteristics	No. of patients	FR+CTC (FU/3 mL, median, IQR)	*P*
Pathological subtypes			
ADC	508	11.60 (9.30, 15.80)	0.013
SCC	58	13.80 (10.83, 17.20)	
Tumor staging			
AIS	24	10.45 (8.58, 11.8508)	<0.001
I	373	11.40 (9.20, 15.10)	
II	38	11.95 (9.70, 21.33)	
III	30	12.75 (10.00, 15.50)	
IV	24	18.10 (12.40, 23.83)	
Lymph node metastasis			
Absent	424	11.40 (9.20, 15.50)	0.165
Present	59	12.30 (9.70, 17.25)	
Degree of invasion			
AIS	24	10.05 (7.88, 11.65)	0.018
MIA	312	10.85 (9.08, 14.35)	
IAC	128	11.90 (9.30, 15.83)	
The number of lesions			
1	385	11.30 (9.00, 15.50)	0.715
>1	38	11.05 (9.48, 13.93)	
Tumor location			
UR	182	11.20 (9.00, 15.13)	0.266
MR	47	10.70 (8.70, 15.30)	
LR	136	12.35 (9.58, 15.53)	
UL	160	11.90 (9.48, 16.05)	
LL	96	12.70 (9.78, 16.63)	
MTD (cm)			
≤1	24	10.70 (8.90, 13.30)	0.014
1–3	188	11.50 (9.10, 16.00)	
3–5	22	11.90 (10.05, 16.75)	
>5	6	14.10 (11.10, 18.13)	

Abbreviations: ADC, adenocarcinoma; AIS: adenocarcinoma in situ; FR+CTC, folate receptor‐positive circulating tumor cell; IAC: invasive adenocarcinoma; LL, lower left; LR, lower right; MIA: minimally invasive adenocarcinoma; MR, middle right; MTD: maximum tumor diameter; SCC, squamous cell carcinoma; UL, upper left; UR, upper right.

**FIGURE 2:**
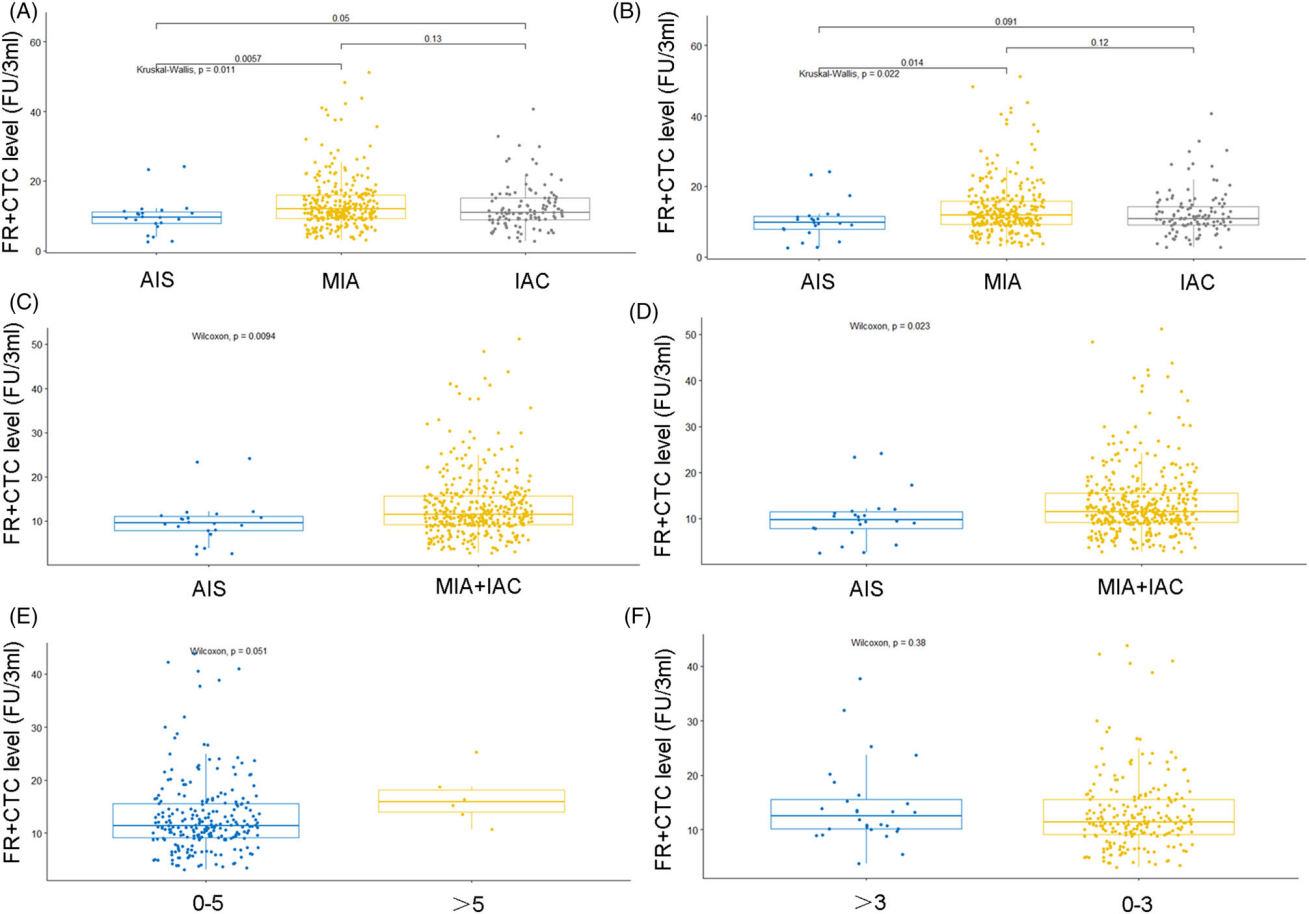
FR+CTC levels of AIS, MIA, and IAC in (A) single lesion and (B) multiple lesions; FR+CTC levels of AIS and MIA + IAC group in (C) single lesion and (D) multiple lesions; (E) FR+CTC levels of 0 < MTD ≤ 5 cm and MTD > 5 cm; (F) FR+CTC levels of 0 < MTD ≤ 3 cm and MTD > 3 cm. AIS, adenocarcinoma in situ; FR+CTC, folate receptor‐positive circulating tumor cell; IAC, invasive adenocarcinoma; MIA, minimally invasive adenocarcinoma; MTD, maximum tumor diameter.

Five hundred eight out of the 653 lung cancer patients with ADC had data on the degree of tumor invasion based on pathological examination of resected specimens. Twenty‐four had AIS, 312 had MIA, and 128 had invasive carcinoma (IAC). The median FR+CTC level in patients with AIS, MIA, and IAC was 10.05 FU/3 mL, 10.85 FU/3 mL, and 11.90 FU/3 mL, respectively; the differences in the FR+CTC level in the three groups were statistically significant (*P* = 0.018) (Table 3). For AIS, MIA and IAC contained only one lesion; FR+CTC levels were related to with the degree of tumor invasion (*P* = 0.011); the *P* value of FR+CTC levels between AIS and IAC is 0.05, and the FR+CTC of AIS patients was significantly lower than MIA (*P* = 0.0057) (Figure 2A). For multiple lesions, the classification was based on the degree of tumor invasion (*P* = 0.022). Further, patients with AIS had significantly lower FR+CTC level than those with MIA (*P* = 0.014) (Figure 2B). The FR+CTC level of AIS patients with single lesion or multiple lesions was significantly lower than group of MIA + IAC with single lesion or multiple lesions (Figure 2C, *P* = 0.0094; Figure 2D, *P* = 0.023).

A total of 240 patients with single lesion in lung cancer group had data on the tumor size before treatment. As shown in Table 3, the median FR+CTC level of group for maximum tumor diameter (MTD) ≤ 1 cm is 10.70 FU/3 mL; for 1 < MTD < 3 cm is 11.50 FU/3 mL; for 3 < MTD < 5 cm is 11.90 FU/3 mL; and for MTD > 5 cm is 14.10 FU/3 mL; the levels of FR+CTC were increased by the increase of MTD, and we can draw a conclusion that there were statistically differences among all groups as the whole *P* value was 0.014. The median FR+CTC level for MTD ≤ 5 cm is lower than MTD > 5 cm, (Figure 2E, *P* = 0.051), so we can infer that MTD (bounded by 5 cm) showed a slight correlation with the FR+CTC level. The median FR+CTC level for MTD ≤ 3 cm is lower than MTD > 3 cm, (Figure 2F, *P* = 0.38).

The median FR+CTC of patients without lymph node metastasis was 11.40 FU/3 mL, which was lower than that of patients with lymph node metastasis (12.30 FU/3 mL); however, there was no significant correlation between the FR+CTCs levels and lymph node metastasis (*P* = 0.165), and there was no significant correlation between FR+CTC and the number of lesions or tumor location (*P* = 0.715 and 0.266, respectively) (Table 3).

## 4 DISCUSSION

In the past, the method of diagnosis of suspected lung cancer relied on chest X‐ray and sputum cytology. However, it has been approved that these two methods cannot be used effectively to improve the survival rate of patients with lung cancer.^21^ Low‐dose computed tomography is recommended for early diagnosis of lung cancer in high‐risk groups, but its high false positive rate can lead to inappropriate surgery,^22^ and it is harmful to patients who are exposed to radiation.^23^, ^24^ Tissue biopsy, as the gold standard for cancer diagnosis, can determine the tumor subtype, stage, and subsequent surgical treatment, but such pathological examination requires tissue excision or resection, which is limited by space and time. Liquid biopsy is an emerging technique and may supplement imaging and serum biomarkers for early diagnosis. It requires multiple methods such as CTCs detection to comprehensively judge the nature of the nodule.^25^ Although CTCs can be released into the blood in the early stage of tumor, there is extensive epithelial‐mesenchymal transition in lung cancer, and tumor cells lose epithelial markers, rendering most CTCs detection methods on the market ineffective.^26^ Therefore, the development of CTCs detection technology for lung tumors is very necessary for early diagnosis, recurrence monitoring, and treatment effect evaluation of lung cancer.

FR is overexpressed in lung cancer cells, but most of the cells in the blood do not express FR (except for active macrophages), which provides ideal conditions for the detection of lung cancer blood targeting FR+CTC. In lung cancer patients, each CTC can express 100 000 orders of folic acid receptors, and PCR amplification can achieve two‐stage signal amplification, so a very small amount of CTCs can be detected in a 3‐mL blood sample.^27^

In our study, we found that the median FR+CTC value of lung cancers was higher than that of the noncancer participants. These findings are consistent with previous research in comparison of FR+CTC levels in benign and malignant groups.^28^, ^29^ We compared the diagnostic value between FR+CTC and tumor biomarkers like NSE, CEA, CA125, CA153, CYFRA21‐1, SCC, and FR+CTC combined with traditional biomarkers. The results showed that FR+CTC levels exhibited the best performance, in terms of sensitivity, in differentiating patients with lung cancer from noncancer participants compared with traditional biomarkers. In the early detection of lung cancer, the sensitivity of FR+CTC is more than three to five times higher than other conventional tumor markers in clinical practice.^30^, ^31^, ^32^ When FR+CTC was combined with NSE, CEA, CA125, CA153, and CYFRA21‐1, the performance improved significantly.

We found that some clinical factors are associated with FR+CTC levels, FR+CTC levels were related to with tumor staging, and as the progression of tumor stage, the level of FR+CTC also increased, which was statistically significant. For multiple lesions, FR+CTC levels were related to with the degree of tumor invasion both in multiple lesions and single lesion. A recent study of 3798 patients from the pulmonary hospital literature analyzed the correlation between FR+CTC and tumor gene expression, stage, and degree of invasion, confirming that the determination of FR+CTC level is a simple and time‐saving method to improve the diagnosis of pulmonary nodules.^33^

We then detected the correlation of FR+CTC levels with pathological subtypes; we found that the median FR+CTC level in SCC group was higher than the group of ADC, which was similar to the previous studies.^34^ What accounts for the difference between the two pathological subtypes? There are some studies, but there are no firm conclusions. Some hold the opinion that the expressions of folate receptor‐α are significantly different between ADC and SCC,^27^, ^35^, ^36^ whereas some thought that it is strongly expressed in both ADC and SCC.^37^

Then, the clinical factors of tumor location were considered, and there was not a statistical difference between the two groups; however, other study concludes that FR+CTC was correlated with tumor location, and it confirmed that the combination of CTCs and CEA can help guide the management of patients with solitary pulmonary nodules suspected of being lung cancer.^38^ Their results demonstrated that CTCs are feasible diagnostic biomarkers in patients with solitary pulmonary nodules, especially in the upper lobe. Furthermore, CTCs combined with CEA showed higher diagnostic efficacy in the upper lobe, subsolid nodules, and nodules ≥8 mm. It may be related to the sample size of the subjects.

Then, the clinical factors of MTD were considered, the levels of FR+CTC were increased by the increase of MTD, and we can draw a conclusion that there were statistically differences among all groups. The median FR+CTC level for MTD ≤ 5 cm is lower than MTD > 5 cm, so we can infer that FR+CTC detection before treatment combined with the MTD can be used to evaluate the subnodular infiltrates less than or equal to 5 cm before surgery, which can guide the clinical operation. According to the clinical data of a pulmonary nodules article,^39^ preoperative CTC detection combined with the MTD can effectively evaluate the subnodular infiltrates ≤2 cm before surgery, which can guide the clinical operation.

Several limitations of the present study should be mentioned. First, even though the sample size was much larger than others, the number of different subgroups varied greatly, which increased uncertainty. Second, our study is single hospital‐based, and it only represents the same source population rather than all population.

FR+CTC combined with conventional tumor markers is a reliable biomarker for the diagnosis of lung cancer. Notably, some clinical factors indeed of tumor staging and degree of invasion, pathological subtypes for ADC and SCC, and MTD (bounded by 5 cm) showed a slight correlation with the FR+CTC level, and more researches are needed.

## AUTHOR CONTRIBUTIONS

Conception and design: Yongping Lin; Administrative support: Yunjian Xu; Provision of study materials or patients: Qianjun Li; Collection and assembly of data: Zhijian Lin; Data analysis and interpretation: Yunjian Xu; Manuscript writing: All authors; Final approval of manuscript: All authors.

## CONFLICT OF INTERESTS STATEMENT

The authors declare that there is no conflict of interests.

## ETHICS STATEMENT

The study was approved by the Research Ethics Committee of the First Affiliated Hospital of Guangzhou Medical University. All patients provided written informed consent before inclusion into this study.

## Data Availability

The datasets used and/or analyzed during the current study are available from the corresponding author on reasonable request.
